# Exploring the impact of growth mindset on psychological symptoms in students from ethnic regions of China: how meaning in life makes a difference

**DOI:** 10.3389/fpsyt.2025.1520645

**Published:** 2025-02-12

**Authors:** Zuozhi Fang, Zhongfang Fu

**Affiliations:** School of Psychological and Cognitive Sciences, Beijing Key Laboratory of Behavior and Mental Health, Key Laboratory of Machine Perception (Ministry of Education), Peking University, Beijing, China

**Keywords:** growth mindset, meaning in life, mental health, ethnic regions, disadvantaged adolescents

## Abstract

**Introduction:**

Drawing on implicit belief theory, this study examines the relationship between growth mindset and psychological symptoms in adolescents from ethnic minority regions in China, with a focus on the mediating role of meaning in life. Understanding this mechanism can provide insights into protective factors that support adolescent mental health.

**Methods:**

Study 1 employed a cross-sectional mediation model with 1,184 middle school students from Yunnan and Guangxi, using the Growth Mindset Scale, Meaning in Life Questionnaire, and Brief Symptom Inventory. Study 2 adopted a longitudinal mediation model, tracking 618 students over eight months. Multiple regression and structural equation modeling were used to assess direct and indirect effects, controlling for socioeconomic status and age.

**Results:**

Growth mindset was positively associated with meaning in life and negatively associated with psychological symptoms, including depression, anxiety, interpersonal sensitivity, and hostility. Longitudinal findings confirmed that growth mindset at T1 predicted lower psychological symptoms at T2, with meaning in life serving as a partial mediator. The mediation effects accounted for 21.55% to 43.33% of the total effect across different symptoms, indicating a cumulative impact over time.

**Discussion:**

These findings highlight the protective role of growth mindset and the importance of meaning in life in adolescent mental health. The consistency of the mediation effect across cross-sectional and longitudinal models suggests that interventions promoting growth mindset and meaning-building strategies could have sustained mental health benefits. These insights have practical implications for school-based programs aimed at fostering resilience and psychological well-being in adolescents.

## 1 Introduction

Adolescents worldwide are increasingly affected by mental health issues, with an estimated 13.4% experiencing significant difficulties (1). Emotional disorders, such as depression and anxiety, are particularly widespread (2), especially in low- and middle-income countries (3). The situation in China mirrors this global trend, as the prevalence of mental disorders has risen steadily over the past 30 years (4). Mental health challenges in China’s ethnic minority regions, in particular, warrant special attention due to their unique socio-cultural contexts. For instance, research has shown that psychological abuse and neglect are more prevalent among girls in ethnic minority regions than in Han regions, contributing to long-term negative mental health outcomes (5). Additionally, ethnic minority students often face difficulties in psychological adjustment and identity development, influenced by factors such as cultural assimilation pressures and socio-economic disparities, which are associated with increased school dropout rates (6). Ethnic minority university students also report heightened levels of psychological stress, including anxiety and depression (7). Existing studies largely attribute these issues to socio-economic challenges, cultural assimilation pressures, and limited access to mental health resources. However, these explanations often focus on external barriers and overlook internal psychological factors, such as individuals’ beliefs and coping mechanisms. For example, growth mindset, which emphasizes the potential for change and personal improvement, may serve as a protective factor, fostering resilience and reducing the impact of stress (8). Furthermore, the role of meaning in life—a key determinant of Psychological symptoms—has been insufficiently explored in this context. In a study conducted in Yunnan, Guizhou, and Guangxi, the detection rates for mild and moderate psychological problems among middle school students were 57.4% and 9.4%, respectively, with severe psychological problems detected in 0.2% of cases (9). These findings underscore the urgent need to investigate mental health issues in ethnic minority regions of China from both socio-cultural and psychological perspectives, focusing on mechanisms such as growth mindset and meaning in life.

According to Dweck (10), a growth mindset is the belief that one’s abilities and intelligence can be developed through dedication, hard work, and the embrace of challenges, productive feedback, and the success of others. Growth Mindset is an implicit belief, individuals with a growth mindset believe they can improve their own qualities and adapt to their surroundings. In contrast, a fixed mindset assumes that traits like intelligence, personality, and even moral values are fixed and unchangeable (10). Existing research highlights that a growth mindset can mitigate mental health problems, whereas a fixed mindset is linked to a greater risk of such issues (11, 12). Studies also suggest that a growth mindset not only helps reduce mental health problems but also fosters a positive “system of meaning” that shapes individuals’ interpretations of challenges and behaviors (13–16). Importantly, research has shown that a growth mindset can positively predict meaning in life (17), suggesting that individuals with a growth mindset are better equipped to derive meaning from stressful situations by reevaluating their self-worth and life goals. Thus, the connection between growth mindset and mental health may be mediated by a stronger sense of meaning in life. This theoretical framework provides a basis for exploring the mediating role of meaning in life in the relationship between growth mindset and mental health.

### 1.1 The relationship between growth mindset and psychological symptoms

According to Dweck’s implicit belief theory (15), individuals can hold either a growth mindset or a fixed mindset. A growth mindset encourages healthy and adaptive responses to anxiety, frustration, and disappointment, fostering greater resilience and persistence in the face of challenges (18). This resilience stems from the use of effort-based strategies when pursuing goals (19). Individuals with a growth mindset are often less stressed and report fewer psychological symptoms. For instance, adolescents facing family stress may experience reduced externalizing problems when equipped with a growth mindset (20).

In contrast, a fixed mindset has been shown to predict higher self-reported stress levels (21), increased anxiety following rejection, and greater psychosocial stress and mental health issues (22, 23). This is particularly relevant when exploring its role in adolescent mental health. A meta-analysis found that adolescents with stronger fixed mindsets exhibited more emotional and behavioral problems, an association consistent across variables such as gender, age, and methodological factors (22). Furthermore, longitudinal research revealed that a stronger fixed mindset in early adolescence predicted more severe mental health problems (both internalizing and externalizing) over a six-month period (24). Based on this, we propose Hypothesis 1: A growth mindset is negatively associated with psychological symptoms.

### 1.2 The mediating role of meaning in life

Steger’s dual-dimension model of meaning in life could provide valuable insights into understanding the association between growth mindset and mental health. Meaning in life refers to an individual’s perception of purpose, mission, and overarching goals in life (25). In this model, two primary dimensions (i.e., presence of meaning, search for meaning) were addressed. The presence of meaning refers to having clear life goals and direction. Individuals with a growth mindset are often more inclined to pursue personal growth and realize their potential, contributing to a heightened sense of purpose. This sense of purpose further contributes to their presence of meaning. This process also aligns closely with the second dimension of meaning in life (i.e., search for meaning). With a growth-oriented mindset, individuals continuously adjust their understanding of meaning and explore new direction and values, which ultimately enrich their lives.

Among middle school students, those with a growth mindset have been found to engage in more positive evaluations of life, which in turn enhances their sense of meaning (17). Evidence from organizational psychology further supports these findings, demonstrating similar effects in adult populations (26). Thus, individuals who hold a growth mindset, and believe in the continuous development of their abilities, are more likely to view life’s challenges as opportunities for growth. This positive outlook encourages adaptive coping strategies, which may strengthen their sense of meaning in life (27, 28).

On the other hand, meaning in life is widely recognized as a critical contributor to psychological symptoms. The sense of purpose, mission, and coherence can serve as a psychological anchor during challenging times. Research demonstrated that individuals who perceive their lives as meaningful report higher levels of positive affect and psychological symptoms (29, 30). Therefore, our second hypothesis is that meaning in life could act as a mediator between growth mindset and psychological symptoms.

Adolescents are facing an important developmental task in which they begin to shape their psychological and social identity. During this stage, the complex questions about their purposes, values, and positions in this world could spur a natural drive to seek meaning. To facilitate this meaning-searching process among adolescents is essential for their positive development and long-term mental health. Therefore, to elucidate the path that whether growth mindset could foster meaning in life becomes crucial for potential intervention.

**Figure 1:**
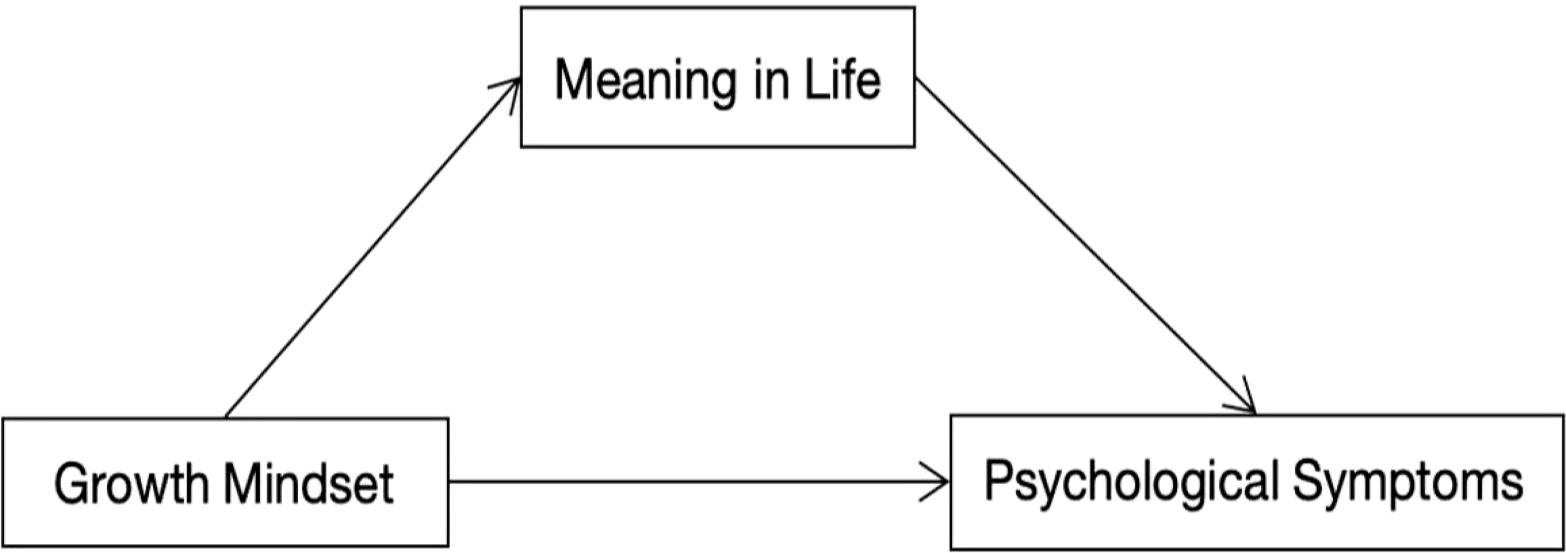
The theoretical model diagram of the relationship between growth mindset and psychological symptoms.

Although previous research has established independent associations among growth mindset, meaning in life, and psychological symptoms, the exact pathways remain insufficiently clarified. Driven by these concerns, the current study aimed to examine the relationships among these three constructs as mediation model in both cross-sectional and longitudinal datasets to cross-validate the mediation model below (see **Figure 1**) and provides a comprehensive examination of the mechanisms linking growth mindset and mental health.

Study 1 conducted an initial exploration of the proposed relationships using cross-sectional data, which enabled us to pinpoint significant associations and establish a foundational framework for the mediation model. This initial evidence informed the design and interpretation of Study 2, in which longitudinal data were employed to examine the temporal dynamics of the model. Essentially, the findings from Study 1 provided preliminary validation of the theoretical connections, ensuring that the subsequent longitudinal study was built on empirically supported associations.

## 2 Study 1: the concurrent relationship between growth mindset and psychological symptoms with meaning in life as mediator

### 2.1 Participants and procedures

A convenience sampling approach was employed to collect data from middle school students at three schools in Yunnan and Guangxi. In this study, 60.56% of the participants identified as ethnic minorities. Inclusion Criteria: Participants were full-time students enrolled in the selected middle schools located in ethnic regions and received approval from the school authorities to participate. All participants demonstrated sufficient language proficiency to understand and complete the questionnaires. Exclusion Criteria: Students who were absent or unwilling to participate at the time of data collection, and those with incomplete or invalid responses were excluded. Additionally, participants exhibiting severe cognitive or communication difficulties that hindered their understanding of the survey materials were not included. A total of 1,200 questionnaires were distributed, and 1,184 valid responses were returned, resulting in a high response rate of 98.67%. The sample comprised 616 males (52.5%) and 556 females (47.4%), with 9 missing cases (0.1%). In terms of residential background, 553 students were from urban areas (46.7%) and 612 from rural areas (51.7%), with 18 missing cases (1.6%). Ethnically, 454 students identified as Han (38.34%) and 717 as ethnic minorities (60.56%), with 12 missing cases (1.1%). The participants’ mean age was 16.26 years (*SD* = 0.90).

This study was conducted in accordance with the ethical standards outlined in the Declaration of Helsinki and was approved by the Institutional Review Board (IRB) of Guangxi Normal University. Prior to participation, all participants were provided with a comprehensive description of the study’ s purpose, procedures, and potential risks. Informed consent was obtained from all participants, who were assured of their right to withdraw at any time without penalty and the confidentiality of their responses. Data collected was anonymized and stored securely to protect participant privacy.

### 2.2 Measures

#### 2.2.1 Growth mindset scale

The Growth Mindset Scale developed by Blackwell et al. (31) was used in this study. The scale includes six items, such as “Your intelligence is something about you that you can’t change,” rated on a 6-point Likert scale (1 = “Strongly disagree” to 6 = “Strongly agree”). Three of the items are reverse scored. A higher overall average score indicates a higher level of growth mindset. In this study, the scale demonstrated good internal consistency, with a Cronbach’s alpha of 0.869.

#### 2.2.2 Meaning in life questionnaire

The Chinese version of the Meaning in Life Questionnaire (MLQ), developed by Steger et al. (25) and revised by Wang and Dai (32), was used to assess participants’ sense of meaning in life. The scale consists of two dimensions: Presence of Meaning (e.g., “I have found a satisfying purpose in life”) and Search for Meaning (e.g., “I am seeking meaning in my life”). It includes 10 items, scored on a 7-point scale (1 = “Strongly disagree” to 7 = “Strongly agree”), with item 9 reverse scored. Higher average scores reflect a stronger sense of meaning in life. Previous research has confirmed the scale’s reliability and validity among middle school students (33). In the current study, the Cronbach’s alpha for the MLQ was 0.844.

#### 2.2.3 Psychological symptoms

Psychological symptoms were measured using four subscales from the Brief Symptom Inventory (BSI) (34): Depression, Anxiety, Interpersonal Sensitivity, and Hostility, with a total of 21 items. Responses were rated on a 5-point scale (0 to 4). In this study, the overall internal consistency for psychological symptoms was excellent, with a Cronbach’s alpha of 0.939. The Cronbach's alphas of the subscales were also adequate: Depression (α = 0.827), Anxiety (α = 0.872), Interpersonal Sensitivity (α = 0.763), and Hostility (α = 0.773).

#### 2.2.4 Socioeconomic status

Socioeconomic status (SES) was assessed using the MacArthur Ladder Scale (35), in which participants rate their perceived social standing on a 1–10 scale, where 1 indicates the lowest position and 10 indicates the highest. This scale measures subjective perceptions of income, educational attainment, and occupational prestige. Higher scores reflect a higher perceived socioeconomic status, while lower scores indicate a lower subjective SES. The scale demonstrated acceptable reliability in this study (Cronbach's alpha = 0.720).

### 2.3 Data Processing and Analysis

Data preprocessing, descriptive statistics, and correlation analyses were performed using SPSS 27.0. Multiple regression analyses were conducted using SPSS, and the standard errors and 95% confidence intervals for the indirect effects were computed.

### 2.4 Results

#### 2.4.1 Common method bias test

Since all data were collected via self-report questionnaires, common method bias was a potential concern. To mitigate this, we emphasized anonymity during data collection, reverse-scored some items, and used validated scales with high reliability. *Post hoc* analysis using Harman’s single-factor test revealed eight factors with eigenvalues greater than 1, with the first factor accounting for 26.02% of the variance, which is well below the 40% threshold. These results suggest that common method bias is not a significant concern in this study.

#### 2.4.2 Descriptive statistics and correlation analysis

As presented in **Table 1**, growth mindset was positively correlated with meaning in life (*p* < 0.001) and negatively correlated with psychological symptoms (*p*< 0.001), including depression, anxiety, interpersonal sensitivity, and hostility. Meaning in life was also negatively correlated with depression, anxiety, interpersonal sensitivity, and hostility (*p*< 0.001). Since socioeconomic status was significantly correlated with the key variables (*p*< 0.001), it was controlled in subsequent analyses. It is worth noting that the non-significant correlation between ethnic minority status and psychological symptoms, may be due to the multifaceted nature of mental health, with psychological symptoms representing only one dimension. Additionally, ethnic minority adolescents face unique mental health risks, and the tools used may not fully capture culturally specific expressions of distress or unique psychological challenges.

**Table 1:** Descriptive statistics and correlations among the research variables (*n* = 1184).

Variable	1	2	3	4	5	6	7	8	9	10	11	12	13
1. Age	—												
2. Gender	—	—											
3. Family location	—	—	—										
4. Ethnic minority	—	—	—	—									
5. SES (individual)	—	—	—	—	—								
6. SES (family)	—	—	—	—	—	—							
7. Growth Mindset	0.22^***^	-0.04	0.10^**^	-0.19^***^	0.12*	0.12*	—						
8. Meaning in Life	-0.17^***^	0.01	-0.09^**^	0.09^**^	0.17**	0.17*	0.14^***^	—					
9. Psychological Symptoms	0.05	-0.01	0.02	-0.04	-0.24^***^	-0.22^***^	-0.10^**^	-0.28^***^	—				
10. Depression	0.07*	0.01	0.03	0.04	-0.25^***^	-0.22^***^	-0.11^***^	-0.30^***^	0.92^***^	—			
11. Anxiety	-0.01	0.01	0.02	-0.02	-0.21^***^	-0.18^***^	-0.08^**^	-0.24^***^	0.91^***^	0.80^***^	—		
12. Interpersonal Sensitivity Interpersonal Sensitivity	0.05	0.01	0.03	-0.04	-0.22^***^	-0.20^***^	-0.09^**^	-0.24^***^	0.89^***^	0.77^***^	0.74^***^	—	
13. Hostility	0.05	0.02	0.02	-0.05	0.14^**^	-0.14^**^	-0.09^**^	-0.22^***^	0.84^***^	0.69^***^	0.70^***^	0.69^***^	—
*M*	16.26	—	—	—	4.92	5.67	3.56	4.71	1.01	0.93	0.96	1.15	1.06
*SD*	0.90	—	—	—	1.94	1.54	1.15	1.07	0.80	0.85	0.91	0.88	0.98
*Score Range*	—	—	—	—	—	—	1 - 6	1 - 7	0 - 4	0 - 4	0 - 4	0 - 4	0 - 4

*p < 0.05, **p < 0.01,***p < 0.001. Categorical variables are dummy coded: Gender (Male = 1, Female = 0); Family location (Urban = 0, Rural = 1); Ethnic minority (Yes = 1, No = 0).

#### 2.4.3 The mediating role of meaning in life

Using Model 4 from the Process 4.1 macro, we tested the mediating effect of meaning in life on the relationship between growth mindset and psychological symptoms. All variables were standardized before analysis, age and socioeconomic status (SES) were controlled as demographic variables. Growth mindset was entered as the independent variable, meaning in life as the mediator, and psychological symptoms and their subdimensions as the dependent variables (see **Table 2**).

**Table 2:** The mediation effects from growth mindset to psychological symptoms (total scale and four subscales) in cross-sectional dataset.

Path	*β*	*SE*	*t*	*p*	*95%Cl*
Model 1 (Overall psychological symptoms)					
a path	0.13	0.03	5.01	<0.001	[0.081,0.186]
b path	-0.21	0.02	-9.61	<0.001	[-0.240,-0.157]
Direct effect (c’)	-0.04	0.02	-2.18	0.029	[-0.081,-0.004]
Total effect (c)	-0.07	0.02	-3.48	<0.001	[-0.109,-0.031]
Indirect effect	-0.03	0.01	——	——	[-0.041,-0.014]
Model 2 (Depression)					
a path	-0.13	0.03	5.01	<0.001	[0.081,0.186]
b path	-0.23	0.02	-10.49	<0.001	[-0.277,-0.189]
Direct effect (c’)	-0.05	0.02	-2.55	0.010	[-0.093,-0.012]
Total effect (c)	-0.08	0.02	-3.93	<0.001	[-0.126,-0.042]
Indirect effect	-0.03	0.07	——	——	[-0.043,-0.016]
Model 3 (Anxiety)					
a path	0.13	0.03	5.01	<0.001	[0.081,0.186]
b path	-0.20	0.02	-8.05	<0.001	[-0.244,-0.148]
Direct effect (c’)	-0.03	0.02	-1.52	0.1297	[-0.079,0.010]
Total effect (c)	-0.06	0.02	-2.64	0.009	[-0.105,-0.015]
Indirect effect	-0.03	0.01	——	——	[-0.039,-0.013]
Model 4 (Interpersonal Sensitivity)					
a path	0.13	0.03	5.01	<0.001	[0.081,0.196]
b path	-0.19	0.02	-8.04	<0.001	[-0.235,-0.143]
Direct effect (c’)	-0.04	0.02	-1.99	0.047	[-0.086,-0.001]
Total effect (c)	-0.07	0.02	-3.10	0.002	[-0.112,-0.025]
Indirect effect	-0.02	0.01	——	——	[-0.037,-0.012]
Model 5 (Hostility)					
a path	0.13	0.03	5.01	<0.001	[0.081,0.186]
b path	-0.20	0.03	-7.49	<0.001	[-0.248,-0.145]
Direct effect (c’)	-0.05	0.02	-2.07	0.034	[-0.098,-0.003]
Total effect (c)	-0.08	0.02	-3.11	0.002	[-0.125,-0.028]
Indirect effect	-0.03	0.007	——	——	[-0.040,-0.012]

**Figure 2:**
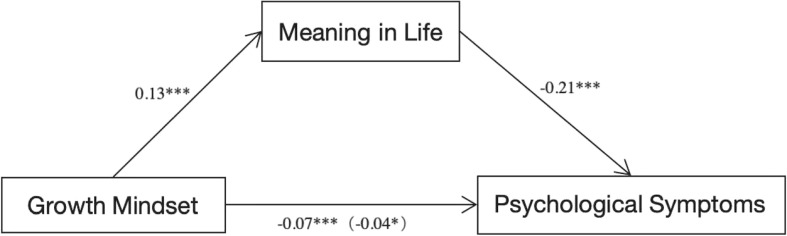
The path analysis of mediation effects from growth mindset to psychological symptoms in the cross-sectional dataset. * denotes statistical significance at the 0.05 level and *** denotes statistical significance at the 0.001 level.

In this cross-sectional dataset, we explored how growth mindset predicts psychological symptoms, with meaning in life as a mediator. Using the Bootstrap method, we calculated the standard errors and 95% confidence intervals of the indirect effects. The results revealed that growth mindset significantly predicted psychological symptoms, both directly and indirectly through meaning in life. Specifically, growth mindset was a significant predictor of meaning in life (*β*= 0.13, *t* = 5.01, *p*< 0.001), which in turn predicted lower psychological symptoms (*β*= -0.21, *t*= -9.61, *p*< 0.001). The direct effect of growth mindset on psychological symptoms was significant (*β*= -0.07, *t* = -3.48, *p*< 0.001), but weakened after accounting for meaning in life (*β* = -0.04, *t* = -2.18, *p* = 0.029), indicating partial mediation. The indirect effect (*β*= -0.03, 95% *CI* [-0.036, -0.013]) accounted for 37.14% of the total effect, supporting the study’s hypothesis (see **Figure 2**).

Further we replicated this analysis using depression, anxiety, interpersonal sensitivity, and hostility as outcome separately. In the depression model, the indirect effect of growth mindset through meaning in life was significant (*β*= -0.03, 95% *CI* [-0.047, -0.017]), representing 37.38% of the total effect. In the anxiety model, growth mindset’s direct effect was not significant (*β* = -0.03, *p* = 0.13), indicating full mediation, with an indirect effect of *β*= -0.03, 95% *CI* [-0.041, -0.014], accounting for 43.33% of the total effect. In the interpersonal sensitivity and hostility models, the indirect effects were *β* = -0.03, 95% *CI*[-0.040, -0.015] and *β*= -0.03, 95% *CI*[-0.040, -0.014], accounting for 35.29% and 32.50% of the total effect, respectively. These findings suggest that meaning in life significantly mediates the relationship between growth mindset and various psychological symptoms (see **Table 2**).

The results of Study 1 indicate that growth mindset is significantly associated with both meaning in life and psychological symptoms, with meaning in life serving as a mediator in this relationship. These findings provide preliminary support for Hypotheses 1 and 2. However, due to the cross-sectional nature of this study, causal inferences are limited. To strengthen causal interpretations, Study 2 will employ a longitudinal design, enabling a more robust examination of these hypotheses.

## 3 Study 2: the temporal sequence of effects from growth mindset on psychological symptoms

### 3.1 Participants and procedures

A cluster sampling method was used to recruit 620 students from four middle schools in Guangxi and Yunnan for this study. Data were collected at two time points, with an eight-month interval between measurements. Our sample reflects the regional diversity, with 41.2% of participants identifying as ethnic minorities. Inclusion Criteria: Participants were full-time students enrolled in the selected middle schools located in ethnic regions and received approval from the school authorities to participate. All participants demonstrated sufficient language proficiency to understand and complete the questionnaires. Exclusion Criteria: Students who were absent or unwilling to participate at the time of data collection, and those with incomplete or invalid responses were excluded. Additionally, participants exhibiting severe cognitive or communication difficulties that hindered their understanding of the survey materials were not included. To minimize familiarity bias from repeated testing, the order of the scales was randomized at each administration. The first round of data collection took place in March 2023 (T1). After removing invalid questionnaires and handling missing data, 618 valid responses were retained, consisting of 310 males and 308 females. The second round was conducted in December 2023 (T2), during which 30 participants were lost due to reasons such as illness or transferring schools, resulting in an attrition rate of 4.9%. Ultimately, 588 participants completed both assessments, with an average age of 12.73 ± 0.82 years. Among them, 277 were male (47.2%), 295 were female (50.3%), and 16 did not report their gender (2.7%). In terms of family structure, 146 participants (24.9%) were only children, 427 (72.7%) were non-only children, and 14 (2.4%) did not report their family structure. Additionally, 242 participants (41.2%) identified as ethnic minorities, 328 (55.9%) were non-minorities, and 17 (2.9%) did not report their ethnicity.

This study was conducted in accordance with the ethical standards outlined in the Declaration of Helsinki and was approved by the Institutional Review Board (IRB) of Guangxi Normal University. Prior to participation, all participants were provided with a comprehensive description of the study’s purpose, procedures, and potential risks. Informed consent was obtained from all participants, who were assured of their right to withdraw at any time without penalty and the confidentiality of their responses. Data collected was anonymized and stored securely to protect participant privacy.

### 3.2 Measures

#### 3.2.1 Growth mindset

The Growth Mindset Scale (GMS) developed by Chen et al. (36) was used to measure participants’ growth mindset. The scale consists of 18 items, with four reverse-scored items, rated on a 5-point Likert scale (1 = “Strongly disagree” to 5 = “Strongly agree”). Higher scores indicate stronger growth mindset. The Cronbach’s alpha for the scale was 0.861 at T1 and 0.852 at T2.

#### 3.2.2 Meaning in life

The Meaning in Life scale used in Study 1 was also employed here. In this study, the Cronbach’s alpha was 0.842 at T1 and 0.816 at T2. For the subdimensions, the alphas for Presence of Meaning were 0.641 (T1) and 0.729 (T2), and for Search for Meaning, 0.863 (T1) and 0.827 (T2).

#### 3.2.3 Psychological symptoms

The Psychological Symptoms scale from Study 1 was used again in this study. The Cronbach’s alpha was 0.959 at T1 and 0.964 at T2, indicating high reliability.

### 3.3 Data processing and analysis

Data preprocessing, descriptive statistics, and correlation analyses were performed using SPSS 27.0. Multiple regression analyses were conducted using Python, and the Delta Method was employed to compute the standard errors and 95% confidence intervals for the indirect effects. To validate the robustness of the findings, structural equation modeling (SEM) was also applied as a supplementary method.

### 3.4 Results

#### 3.4.1 Common method bias test

Considering that the Growth Mindset Scale, Meaning in Life Scale, and Brief Symptom Inventory were self-report measures, there was potential for common method bias. To mitigate this, the study employed several strategies: (1) to minimize within-test bias, we balanced the order of items, mixed positively and negatively worded items, and ensured anonymity to reduce response biases. (2) to address between-test bias, we spaced the two rounds of data collection eight months apart and conducted them in different locations. (3) Harman’s single-factor test revealed 8 and 7 factors with eigenvalues greater than 1 in the two rounds of data collection, with the first factor explaining 29.05% and 28.71% of the variance, respectively, both below the 40% threshold. This analysis confirmed that common method bias was not a significant issue in the study.

#### 3.4.2 Descriptive statistics and correlation analysis

As shown in **Table 3**, growth mindset and meaning in life were significantly positively correlated at both time points, while growth mindset and meaning in life were both negatively correlated with psychological symptoms, showing consistent results across time.

**Table 3:** Descriptive statistics and correlations among the variables in the mediation model (*n* = 618).

Variable	*M* ± *SD*	T1 Growth mindset	T2 Growth mindset	T1 Meaning in Life	T2 Meaning in Life	T1 Psychological Symptoms	T2 Psychological Symptoms
1. T1 Growth mindset	3.98 ± 0.57	—					
2. T2 Growth mindset	3.58 ± 0.59	0.27^***^	—				
3. T1 Meaning in Life	4.73 ± 1.17	0.35^***^	0.17^***^	—			
4. T2 Meaning in Life	4.78 ± 1.10	0.25^***^	0.38^***^	0.28^***^	—		
5. T1 Psychological Symptoms	0.91 ± 0.85	-0.40^***^	-0.14^***^	-0.29^***^	-0.16^***^	—	
6. T2 Psychological Symptoms	1.04 ± 0.92	-0.22^***^	-0.19^***^	-0.12**	-0.26^***^	0.40^***^	—

** denotes statistical significance at the 0.01 level and *** denotes statistical significance at the 0.001 level.

#### 3.4.3 Longitudinal mediation analysis of growth mindset and psychological symptoms

**Figure 3:**
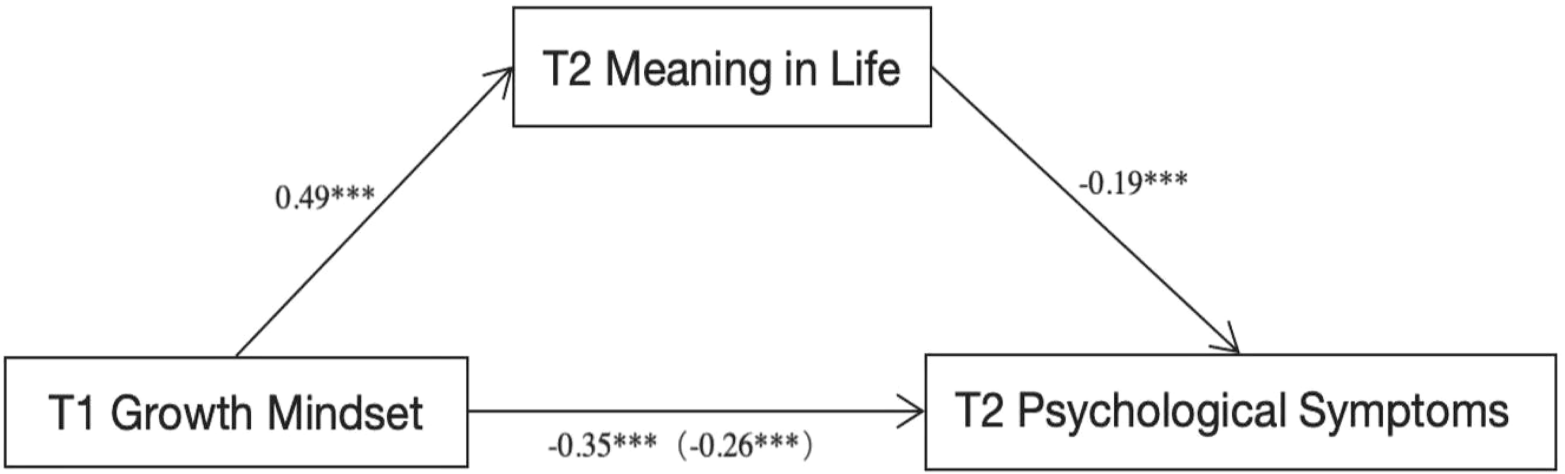
The path analysis of mediation effects from growth mindset to psychological symptoms in the longitudinal tracking dataset. *** denotes statistical significance at the 0.001 level.

In this analysis, growth mindset at T1 was the independent variable, psychological symptoms at T2 (including overall symptoms, depression, anxiety, interpersonal sensitivity, and hostility) were the dependent variables, and meaning in life at T2 served as the mediator. Age and family monthly income were controlled as demographic variables. The Delta Method was used to compute standard errors and 95% confidence intervals for the indirect effects. The findings revealed that growth mindset at T1 significantly predicted psychological symptoms at T2, both directly and indirectly through meaning in life (see **Figure 3**). Specifically, growth mindset significantly predicted meaning in life (*β*= 0.49, *t*= 6.29, *p*< 0.001), which in turn significantly predicted psychological symptoms (*β* = -0.19, *t* = -5.51, *p* < 0.001). The direct effect of growth mindset on psychological symptoms was significant (*β*= -0.35, *t*= -5.34, *p*< 0.001), but this effect weakened after controlling for meaning in life (*β*= -0.26, *t*= -3.91, *p*= 0.0001), indicating a partial mediation effect. The indirect effect was *β* = -0.09, 95% *CI* [-0.132, -0.053], accounting for 26.19% of the total effect. These results suggest that meaning in life significantly mediates the relationship between growth mindset and psychological symptoms, supporting the study’s hypothesis. The results from SEM were highly consistent with those obtained through regression, with nearly identical estimates for the mediation paths (see details in the **Supplementary Materials**).

Similar patterns were observed in the models with depression, anxiety, interpersonal sensitivity, and hostility as outcomes separately (see **Table 4**). In the depression model, the indirect effect of growth mindset through meaning in life was significant (*β*= -0.11, 95% *CI*[-0.165, -0.047]), accounting for 29.81% of the total effect. In the anxiety model, the indirect effect was *β*= -0.08, 95% *CI* [-0.121, -0.042], accounting for 27.42% of the total effect. In the interpersonal sensitivity and hostility models, the indirect effects were *β*= -0.09, 95% *CI*[-0.126, -0.046] and *β*= -0.10, 95% *CI* [-0.135, -0.056], accounting for 21.55% and 25.72% of the total effects, respectively. These findings further support the mediating role of meaning in life in the relationship between growth mindset and various psychological symptoms.

**Table 4:** The mediation effects from growth mindset to psychological symptoms (total scale and four subscales) in the longitudinal tracking model.

Path	*β*	*SE*	*t*	*p*	*95%Cl*
Model 1 (Brief Symptom)					
a path	0.49	0.08	6.29	<0.001	[0.338,0.645]
b path	-0.19	0.03	-5.51	<0.001	[-0.225,-0.121]
Direct effect (c’)	-0.26	-0.07	-3.91	<0.001	[-0.482,-0.223]
Total effect (c)	-0.35	0.07	-5.34	<0.001	[-0.391,-0.129]
Indirect effect	-0.09	0.02	——	——	[-0.036,-0.129]
Model 2 (Depression)					
a path	0.49	0.08	6.29	<0.001	[0.338,0.645]
b path	-0.22	0.03	-6.18	<0.001	[-0.284,-0.147]
Direct effect (c’)	-0.25	0.07	-3.66	<0.001	[-0.383,-0.116]
Total effect (c)	-0.36	0.07	-5.23	<0.001	[-0.489,-0.222]
Indirect effect	-0.11	0.02	——	——	[-0.047,-0.017]
Model 3 (Anxiety)					
a path	0.49	0.08	6.29	<0.001	[0.338,0.645]
b path	-0.17	0.04	-4.36	<0.001	[-0.241,-0.091]
Direct effect (c’)	-0.22	0.07	-2.91	0.004	[-0.362,0.070]
Total effect (c)	-0.30	0.07	-4.08	<0.001	[-0.441,-0.154]
Indirect effect	-0.08	0.02	——	——	[-0.041,-0.014]
Model 4 (Interpersonal Sensitivity)					
a path	0.49	0.08	6.29	<0.001	[0.338,0.645]
b path	-0.17	0.04	-4.81	<0.001	[-0.246,-0.645]
Direct effect (c’)	-0.31	0.07	-4.41	<0.001	[-0.452,-0.173]
Total effect (c)	-0.40	0.07	-5.70	<0.001	[-0.536,-0.261]
Indirect effect	-0.09	0.02	——	——	[-0.040,-0.015]
Model 5 (Hostility)					
a path	0.49	0.08	6.29	<0.001	[0.338,0.645]
b path	-0.19	0.04	-5.23	<0.001	[-0.248,-0.122]
Direct effect (c’)	-0.28	0.07	-3.80	<0.001	[-0.419,-0.134]
Total effect (c)	-0.37	0.07	-5.17	<0.001	[-0.514,-0.231]
Indirect effect	-0.10	0.02	——	——	[-0.041,-0.014]

## 4 Discussion

This study, grounded in implicit theory, examines the influence of growth mindset on the mental health of middle school students in ethnic minority regions of China, with a focus on the mediating role of meaning in life. Both the regression with bootstrap and SEM model consistently revealed the mediation effect, highlighting the reliability of the observed associations and reinforcing the validity of the theoretical framework underlying the model.

In our study, the higher representation of ethnic minority students in the study can be explained by demographic patterns in Guangxi and Yunnan, where minorities account for 37.52% and 33.12% of the population, respectively. This overrepresentation may be attributed to the inclusion of schools in county-level regions, where minority populations are more concentrated. Furthermore, Han students in these provinces often seek educational opportunities in urban centers or outside these regions, leading to a higher proportion of minority students in the sampled schools. These patterns reflect regional disparities in educational access and provide valuable context for understanding the findings. It is worth noting that the psychological symptoms were not influenced by their ethnic status while differences were observed in growth mindset and meaning of life based on minority status. This may be attributed to the shared school environment, which provides a relatively uniform context for psychological experiences, while belief systems are more strongly shaped by cultural and socioeconomic status. Building on this insight, future research should further explore the potential impact of ethnicity on psychological outcomes within this group.

### 4.1 The effect of growth mindset on psychological symptoms

The study results show a significant negative relationship between growth mindset and psychological symptoms-including depression, anxiety, hostility, and interpersonal sensitivity-highlighting growth mindset as a protective factor. This aligns with previous findings on its protective effects on adolescent mental health and symptoms reduction. For example, research indicates that growth mindset significantly reduces negative emotional outcomes in adolescents, such as depression, anxiety, and externalizing behaviors (37), and positively impacts interpersonal relationships by fostering constructive attitudes in challenging situations, enhancing confidence and social interactions (38).

This relationship holds consistently across four psychological symptoms including depression, anxiety, hostility, and interpersonal sensitivity. This result indicates that a growth mindset not only buffers emotional symptoms but also reduces tendencies toward more aggressive or interpersonal conflict-related behaviors. This aligns with existing literature (39–41) and extends the potential applicability of growth mindset as a versatile tool for psychological intervention. Furthermore, the consistent result across cross-sectional and longitudinal data enhances the credibility of the association suggesting that growth mindset not only offers short-term benefits for mental health but may also serve as a preventive factor in the long run.

### 4.2 The mediating role of meaning in life

The current two studies revealed small to modest associations between growth mindset and psychological symptoms, consistent with findings from most previous research (42). However, variations in the strength of these correlations may be influenced by factors such as demographic characteristics and the measurement instruments used. Future research should further explore potential moderators to better understand the conditions under which these relationships may vary. The stronger direct and indirect effects observed in the longitudinal analysis compared to the cross-sectional analysis highlight the added value of capturing temporal dynamics in psychological research. While cross-sectional studies provide a snapshot of the relationships between variables, they are limited in demonstrating the progression and unfolding of these relationships over time. Longitudinal analysis, on the other hand, allows for a deeper understanding of how constructs such as growth mindset influence psychological symptoms across temporal sequences. In this study, the more pronounced effects in the longitudinal model suggest that the influence of a growth mindset on psychological symptoms may strengthen over time, as adolescents consistently internalize its positive impact. The mediating role of meaning in life further emphasizes this temporal sequence, suggesting that sustained engagement with a growth mindset can foster a deeper and more enduring sense of life purpose, which in turn reduces psychological distress (43, 44). This finding underscores the dynamic nature of psychological processes and aligns with theories suggesting that positive cognitive frameworks require time to fully manifest their benefits.

### 4.3 Clinical implication

The stability of this mediating relationship across both datasets implies that intervention targeting growth mindset and fostering meaning in life could be effective for both immediate and long-term mental health benefits. To reinforce these constructs over time could yield sustained reductions in psychological symptoms. Future mental health programs, especially in middle and high schools, could support the environment to foster growth mindset and facilitate meaning-building work to eventually reduce the psychological symptoms in adolescents. To cultivate a more supportive environment, schools could integrate growth mindset interventions by introducing structured psychological education classes for students and professional development programs for teachers specifically designed to foster growth mindsets. These classes could include curriculum modules that teach students about the principles of growth mindset, how it can be applied in academic and personal contexts, and techniques for overcoming challenges through resilience and perseverance. Similarly, professional development programs for teachers could focus on strategies to encourage a growth mindset in their teaching practices and interactions with students. These coordinated efforts are likely to significantly enhance the impact on students’ psychological well-being and academic performance.

### 4.4 Limitations

Limitations of this study include the use of only two time points, which restricts the ability to fully capture the temporal dynamics of the mediation effect, and the simultaneous measurement of both the mediator (meaning in life) and the outcome (psychological symptoms), potentially introducing common method bias. Future studies could incorporate additional time points to more effectively track changes over time and strengthen causal inferences. The reliance on self-reported data also raises the possibility of social desirability bias, suggesting that a combination of self-report and objective measures could enhance validity. Furthermore, although validated Chinese scales were employed, they may not fully capture the nuances of these constructs within the cultural context of ethnic minority regions. Future research should consider culturally adapted tools to improve both generalizability and relevance. In spite of that the overall direction of the mediation paths were in line with each other, the younger average age of the second sample could contribute to the differences in model effects, as developmental stages could influence the relationship between growth mindset, meaning in life, and psychological symptoms. It would be meaningful for future research to explore the age specific mechanisms in order to instruct more relevant intervention. Finally, while the longitudinal design establishes temporal order, the lack of random assignment or experimental manipulation of growth mindset constrains our ability to completely eliminate potential confounding factors, thus limiting the study’s ability to draw definitive causal conclusions about the relationship between growth mindset and mental health. Future research using randomized controlled designs or targeted interventions could provide more definitive insights into causal mechanisms involved.

## 5 Conclusion

In conclusion, our study provides robust evidence that meaning in life consistently mediates the relationship between growth mindset and psychological symptoms, as observed in both cross-sectional and longitudinal analyses. Our findings open new avenues for targeted, evidence-based mental health strategies, especially among the disadvantages adolescents who are vulnerable to psychological symptoms.

## Data Availability

The original contributions presented in the study are included in the article/**Supplementary Material**. Further inquiries can be directed to the corresponding author.
