# Supplementary material for: Exploring the impact of growth mindset on psychological symptoms in students from ethnic regions of China: how meaning in life makes a difference
Source: Front Psychiatry. 2025 Feb 12;16:1520645. doi: 10.3389/fpsyt.2025.1520645 (PMC11861526; doi:10.3389/fpsyt.2025.1520645)
Supplement: Supplementary file 1 [file DataSheet1.pdf]

These are the results of the SEM model used in Study 2, divided into the Psychological symptoms model and four sub-models: depression, anxiety, hostility, and interpersonal sensitivity.

Psychological symptoms Model

Estimator	ML
Optimization method	NLMINB
Number of model parameters	9
Number of observations	554

Model Test User Model:

Test statistic	0.000
Degrees of freedom	0

Model Test Baseline Model:

Test statistic	89.892
Degrees of freedom	7
P-value	0.000

User Model versus Baseline Model:

Comparative Fit Index (CFI)	1.000
Tucker-Lewis Index (TLI)	1.000

Loglikelihood and Information Criteria:

Loglikelihood user model (H0)	-1533.981
Loglikelihood unrestricted model (H1)	-1533.981
Akaike (AIC)	3085.963
Bayesian (BIC)	3124.817
Sample-size adjusted Bayesian (SABIC)	3096.247

Root Mean Square Error of Approximation:

RMSEA	0.000
90 Percent confidence interval - lower	0.000
90 Percent confidence interval - upper	0.000
P-value H_0: RMSEA <= 0.050	NA
P-value H_0: RMSEA >= 0.080	NA

Standardized Root Mean Square Residual:

SRMR	0.000

Parameter Estimates:

Standard errors	Standard
Information	Expected
Information saturated (h1) model	Structured

Regressions:

	Estimate	Std.Err	z-value	P(>|z|)	ci.lower	ci.upper	Std.lv	Std.all
PsychSymptoms ~								
GrwthMndst (c)	-0.262	0.068	-3.829	0.000	-0.396	-0.128	-0.262	-0.160
Age	-0.025	0.038	-0.674	0.500	-0.099	0.048	-0.025	-0.027
Income	0.017	0.037	0.478	0.633	-0.054	0.089	0.017	0.019
LifeMeaning ~								
GrwthMndst (a)	0.486	0.082	5.945	0.000	0.326	0.646	0.486	0.245
Age	-0.019	0.046	-0.418	0.676	-0.110	0.071	-0.019	-0.017
Income	0.027	0.045	0.589	0.556	-0.062	0.115	0.027	0.024
PsychSymptoms ~								
LifeMeanng (b)	-0.186	0.034	-5.400	0.000	-0.254	-0.119	-0.186	-0.225

Variances:

	Estimate	Std.Err	z-value	P(>|z|)	ci.lower	ci.upper	Std.lv	Std.all
.PsychSymptoms	0.757	0.045	16.643	0.000	0.668	0.846	0.757	0.905
.LifeMeaning	1.151	0.069	16.643	0.000	1.015	1.287	1.151	0.939

Defined Parameters:

	Estimate	Std.Err	z-value	P(>|z|)	ci.lower	ci.upper	Std.lv	Std.all
ab	-0.090	0.023	-3.997	0.000	-0.135	-0.046	-0.090	-0.055
total	-0.352	0.068	-5.179	0.000	-0.485	-0.219	-0.352	-0.215

Depression Model

Estimator	ML
Optimization method	NLMINB
Number of model parameters	9
Number of observations	554

Model Test User Model:

Test statistic	0.000
Degrees of freedom	0

Model Test Baseline Model:

Test statistic 97.712

Degrees of freedom 7

P-value 0.000

User Model versus Baseline Model:

Comparative Fit Index (CFI) 1.000

Tucker-Lewis Index (TLI) 1.000

Loglikelihood and Information Criteria:

Loglikelihood user model (H0) -1545.366

Loglikelihood unrestricted model (H1) -1545.366

Akaike (AIC) 3108.732

Bayesian (BIC) 3147.586

Sample-size adjusted Bayesian (SABIC) 3119.016

Root Mean Square Error of Approximation:

RMSEA 0.000

90 Percent confidence interval - lower 0.000

90 Percent confidence interval - upper 0.000

P-value H_0: RMSEA <= 0.050 NA

P-value H_0: RMSEA >= 0.080 NA

Standardized Root Mean Square Residual:

SRMR 0.000

Parameter Estimates:

Standard errors Standard

Information Expected

Information saturated (h1) model Structured

Regressions:

	Estimate	Std.Err	z-value	P(>|z|)	ci.lower	ci.upper	Std.lv	Std.all
Depression ~								
GrwthMndst (c)	-0.251	0.070	-3.593	0.000	-0.387	-0.114	-0.251	-0.149
Age	-0.024	0.038	-0.628	0.530	-0.099	0.051	-0.024	-0.025
Income	0.034	0.037	0.904	0.366	-0.039	0.107	0.034	0.036
LifeMeaning ~								
GrwthMndst (a)	0.486	0.082	5.945	0.000	0.326	0.646	0.486	0.245
Age	-0.019	0.046	-0.418	0.676	-0.110	0.071	-0.019	-0.017

Income	0.027	0.045	0.589	0.556	-0.062	0.115	0.027	0.024
Depression ~								
LifeMeanng (b)	-0.217	0.035	-6.164	0.000	-0.286	-0.148	-0.217	-0.255

Variances:

	Estimate	Std.Err	z-value	P(>|z|)	ci.lower	ci.upper	Std.lv	Std.all
.Depression	0.789	0.047	16.643	0.000	0.696	0.882	0.789	0.892
.LifeMeaning	1.151	0.069	16.643	0.000	1.015	1.287	1.151	0.939

Defined Parameters:

	Estimate	Std.Err	z-value	P(>|z|)	ci.lower	ci.upper	Std.lv	Std.all
ab	-0.105	0.025	-4.279	0.000	-0.154	-0.057	-0.105	-0.063
total	-0.356	0.070	-5.091	0.000	-0.493	-0.219	-0.356	-0.211

Anxiety Model

Estimator	ML
Optimization method	NLMINB
Number of model parameters	9
Number of observations	554

Model Test User Model:

Test statistic	0.000
Degrees of freedom	0

Model Test Baseline Model:

Test statistic	70.034
Degrees of freedom	7
P-value	0.000

User Model versus Baseline Model:

Comparative Fit Index (CFI)	1.000
Tucker-Lewis Index (TLI)	1.000

Loglikelihood and Information Criteria:

Loglikelihood user model (H0)	-1597.807
Loglikelihood unrestricted model (H1)	-1597.807
Akaike (AIC)	3213.613
Bayesian (BIC)	3252.468
Sample-size adjusted Bayesian (SABIC)	3223.898

Root Mean Square Error of Approximation:

RMSEA	0.000
90 Percent confidence interval - lower	0.000
90 Percent confidence interval - upper	0.000
P-value H_0: RMSEA <= 0.050	NA
P-value H_0: RMSEA >= 0.080	NA

Standardized Root Mean Square Residual:

SRMR	0.000

Parameter Estimates:

Standard errors	Standard
Information	Expected
Information saturated (h1) model	Structured

Regressions:

	Estimate	Std.Err	z-value	P(>|z|)	ci.lower	ci.upper	Std.lv	Std.all
Anxiety ~								
GrwthMndst (c)	-0.216	0.077	-2.823	0.005	-0.367	-0.066	-0.216	-0.120
Age	-0.058	0.042	-1.384	0.166	-0.141	0.024	-0.058	-0.057
Income	0.021	0.041	0.504	0.614	-0.060	0.101	0.021	0.021
LifeMeaning ~								
GrwthMndst (a)	0.486	0.082	5.945	0.000	0.326	0.646	0.486	0.245
Age	-0.019	0.046	-0.418	0.676	-0.110	0.071	-0.019	-0.017
Income	0.027	0.045	0.589	0.556	-0.062	0.115	0.027	0.024
Anxiety ~								
LifeMeanng (b)	-0.165	0.039	-4.280	0.000	-0.241	-0.090	-0.165	-0.182

Variances:

	Estimate	Std.Err	z-value	P(>|z|)	ci.lower	ci.upper	Std.lv	Std.all
.Anxiety	0.953	0.057	16.643	0.000	0.841	1.065	0.953	0.938
.LifeMeaning	1.151	0.069	16.643	0.000	1.015	1.287	1.151	0.939

Defined Parameters:

	Estimate	Std.Err	z-value	P(>|z|)	ci.lower	ci.upper	Std.lv	Std.all
ab	-0.080	0.023	-3.474	0.001	-0.126	-0.035	-0.080	-0.045
total	-0.297	0.076	-3.928	0.000	-0.445	-0.149	-0.297	-0.164

Hostility Model

Estimator	ML
Optimization method	NLMINB
Number of model parameters	9

Number of observations	554

Model Test User Model:

Test statistic	0.000
Degrees of freedom	0

Model Test Baseline Model:

Test statistic	84.461
Degrees of freedom	7
P-value	0.000

User Model versus Baseline Model:

Comparative Fit Index (CFI)	1.000
Tucker-Lewis Index (TLI)	1.000

Loglikelihood and Information Criteria:

Loglikelihood user model (H0)	-1582.183
Loglikelihood unrestricted model (H1)	-1582.183

Akaike (AIC)	3182.367
Bayesian (BIC)	3221.221
Sample-size adjusted Bayesian (SABIC)	3192.651

Root Mean Square Error of Approximation:

RMSEA	0.000
90 Percent confidence interval - lower	0.000
90 Percent confidence interval - upper	0.000
P-value H_0: RMSEA <= 0.050	NA
P-value H_0: RMSEA >= 0.080	NA

Standardized Root Mean Square Residual:

SRMR	0.000

Parameter Estimates:

Standard errors	Standard
Information	Expected
Information saturated (h1) model	Structured

Regressions:

	Estimate	Std.Err	z-value	P(>|z|)	ci.lower	ci.upper	Std.lv	Std.all
Hostility ~								
GrwthMndst (c)	-0.282	0.075	-3.780	0.000	-0.428	-0.136	-0.282	-0.158
Age	-0.021	0.041	-0.506	0.613	-0.101	0.060	-0.021	-0.021
Income	0.006	0.040	0.155	0.877	-0.072	0.084	0.006	0.006
LifeMeaning ~								
GrwthMndst (a)	0.486	0.082	5.945	0.000	0.326	0.646	0.486	0.245
Age	-0.019	0.046	-0.418	0.676	-0.110	0.071	-0.019	-0.017
Income	0.027	0.045	0.589	0.556	-0.062	0.115	0.027	0.024
Hostility ~								
LifeMeanng (b)	-0.188	0.038	-5.011	0.000	-0.262	-0.115	-0.188	-0.210

Variances:

	Estimate	Std.Err	z-value	P(>|z|)	ci.lower	ci.upper	Std.lv	Std.all
.Hostility	0.901	0.054	16.643	0.000	0.795	1.007	0.901	0.914
.LifeMeaning	1.151	0.069	16.643	0.000	1.015	1.287	1.151	0.939

Defined Parameters:

	Estimate	Std.Err	z-value	P(>|z|)	ci.lower	ci.upper	Std.lv	Std.all
ab	-0.091	0.024	-3.831	0.000	-0.138	-0.045	-0.091	-0.051
total	-0.373	0.074	-5.051	0.000	-0.518	-0.228	-0.373	-0.210

Interpersonal Sensitivity Model

Estimator	ML
Optimization method	NLMINB
Number of model parameters	9
Number of observations	554

Model Test User Model:

Test statistic	0.000
Degrees of freedom	0

Model Test Baseline Model:

Test statistic	85.466
Degrees of freedom	7
P-value	0.000

User Model versus Baseline Model:

Comparative Fit Index (CFI)	1.000
Tucker-Lewis Index (TLI)	1.000

Loglikelihood and Information Criteria:

Loglikelihood user model (H0)	-1570.894
Loglikelihood unrestricted model (H1)	-1570.894
Akaike (AIC)	3159.788
Bayesian (BIC)	3198.643
Sample-size adjusted Bayesian (SABIC)	3170.073

Root Mean Square Error of Approximation:

RMSEA	0.000
90 Percent confidence interval - lower	0.000
90 Percent confidence interval - upper	0.000
P-value H_0: RMSEA <= 0.050	NA
P-value H_0: RMSEA >= 0.080	NA

Standardized Root Mean Square Residual:

SRMR	0.000

Parameter Estimates:

Standard errors	Standard
Information	Expected
Information saturated (h1) model	Structured

Regressions:

	Estimate	Std.Err	z-value	P(>|z|)	ci.lower	ci.upper	Std.lv	Std.all
InterpersonalSensitivity ~								
GrwthMndst (c)	-0.313	0.073	-4.286	0.000	-0.456	-0.170	-0.313	-0.179
Age	0.009	0.040	0.226	0.821	-0.070	0.088	0.009	0.009
Income	0.003	0.039	0.080	0.936	-0.074	0.080	0.003	0.003
LifeMeaning ~								
GrwthMndst (a)	0.486	0.082	5.945	0.000	0.326	0.646	0.486	0.245
Age	-0.019	0.046	-0.418	0.676	-0.110	0.071	-0.019	-0.017
Income	0.027	0.045	0.589	0.556	-0.062	0.115	0.027	0.024

InterpersonalSensitivity ~

LifeMeanng (b)	-0.172	0.037	-4.670	0.000	-0.244	-0.100	-0.172	-0.19

Variances:

	Estimate	Std.Err	z-value	P(>|z|)	ci.lower	ci.upper	Std.lv	Std.all
.IntrprsnlSnstv	0.865	0.052	16.643	0.000	0.763	0.967	0.865	0.912
.LifeMeaning	1.151	0.069	16.643	0.000	1.015	1.287	1.151	0.939

Defined Parameters:

	Estimate	Std.Err	z-value	P(>|z|)	ci.lower	ci.upper	Std.lv	Std.all
ab	-0.084	0.023	-3.672	0.000	-0.128	-0.039	-0.084	-0.048
total	-0.397	0.072	-5.493	0.000	-0.538	-0.255	-0.397	-0.227
